# Knowledge and Attitudes of Patients with Rheumatoid Diseases towards Biosimilars

**DOI:** 10.31138/mjr.140323.kaa

**Published:** 2024-12-31

**Authors:** Athanasios Chantzaras, John Yfantopoulos, Katerina Koutsogianni

**Affiliations:** 1 National and Kapodistrian University of Athens, Athens, Greece; 2 PanHellenic Federation of Patients, Parents, Caregivers and Friends of Children with Rheumatic Diseases (RHEUMAZIN), Athens, Greece

**Keywords:** biosimilar, biologic therapy, switching, education, attitudes, rheumatology

## Abstract

**Objective:**

To assess patients’ understanding and attitudes towards biosimilars in rheumatoid diseases in Greece.

**Methods:**

A convenience sample of patients with rheumatoid diseases who were members of the largest rheumatoid patient association (RHEUMAZIN) in Greece was selected for this survey. Data on patients’ knowledge and attitudes towards biosimilars were collected with a web-based questionnaire.

**Results:**

Among the 309 patients, 60.2% were being treated with bio-originator products, 11% with biosimilars and another 28.8% did not know the type of their biologic therapy. Only 43.7% of the respondents reported they had adequate information about biologic treatments. About 47.9% knew what biosimilars are exactly and 81.2% stated that they need more information about them. The most influential patient information sources about biologics were rheumatologists (88.3%), the Internet (45%), and patient associations (40.5%). Only about 55–60% of the participants thought that biosimilars are comparable to their reference products in terms of safety, effectiveness, quality and regulatory requirements. Patients with adequate knowledge about biosimilars were significantly less concerned about switching from their reference products. A higher education level, previous biosimilar treatment experience, having rheumatologists, patient associations, regulatory bodies and the internet as main information sources, being better informed about the disease, biologic therapies and biosimilars, working and having adequate information about biosimilars were univariately associated with a significantly higher likelihood of having a positive attitude towards biosimilars.

**Conclusions:**

There is an urgent need for patient education about biosimilars in rheumatic diseases in Greece to enhance patient knowledge and ensure informed decisions on biosimilar use.

## INTRODUCTION

Rheumatoid diseases, like rheumatoid arthritis and axial spondylarthritis, are autoimmune inflammatory conditions characterised by swelling and tenderness of the joints and degradation of the synovial tissues. These conditions are progressive in nature and can result in debilitating joint pain and deformities to the detriment of patients’ physical function and quality of life if disease control is not obtained.^1^ The introduction of biologic disease-modifying antirheumatic drugs has improved significantly the prognosis of patients with rheumatic inflammatory diseases, as increased disease remission or low disease activity is observed in many patients with uncontrolled disease previously treated with conventional synthetic therapies alone.^1,2^

The use of biologic therapies is becoming increasingly common worldwide.^1^ The high cost of these medicines constitutes an important financial challenge for health systems worldwide, as the medical expenses for rheumatic patients may increase to an unsustainable level.^3,4^ Nevertheless, following the expiration of patents, data protection, and market exclusivity periods of bio-originator medicines, biologic products (termed biosimilars) almost identical to the original innovator drugs can enter the market by other drug manufacturers, with the potential to introduce price competition and enhance patient access.^5–7^ The first biosimilar was approved in the European Union in 2006.^6^

Biologic medicines contain active substances that are complex protein molecules originating from living cells or organisms as opposed to chemically synthesised small-molecule medicines.^8^ This requires a manufacturing process based on biotechnology that is more complex than that of non-biological medicines. The molecular structure can slightly vary from batch to batch within specified limitations as well as between the biosimilar and the originator drug.^2^ Hence, biosimilars are regulated differently than small-molecule generics and regulatory bodies have developed specific guidelines for biosimilar approval.^6,9^ Biosimilars are assessed and authorised by the European Medicines Agency based on a comparability exercise to establish biosimilarity with the bio-originator product.^5^ Biosimilars must be highly similar to the originator medicine and meet the regulatory standards of quality, safety and efficacy to be approved.^6^ Biosimilars are tested in at least one indication of the reference product in phase I and III clinical trials and, if scientifically justified, results may be extrapolated to other indications for which the originator product is approved.^5,10^

Overall, biosimilars appear to be lower-cost alternatives to their reference products, which can broaden treatment options available to patients and improve both the accessibility and sustainability of health systems.^7^ However, despite their cost-saving and patient access improving potential, the uptake of biosimilars is lagging.^11,12^ In particular, the health system in Greece is afflicted with several chronic inefficiencies and, despite the plethora of the reforms during the last decade, its long-term sustainability remains uncertain.^13^ Therefore, more cost-effective treatment options, such as biosimilars and generics, delivering the same clinical value with a lower cost would be at least a partial solution to the financing problems of the Greek pharmaceutical system. Although data for the exact market share of biosimilars in the treatment of rheumatic diseases in Greece are not available, a previous study has estimated that, of all patients that receive therapy with erythropoietin (EPO), only 12% are treated with biosimilars.^14^

Several recent studies have documented the challenges related to patient acceptance of biosimilars. Patients’ concerns about the quality, efficacy and safety of biosimilars compared with the reference products may at least partly explain their low uptake.^8,9^ Negative perceptions towards biosimilars not only exacerbates hesitancy to accept biosimilars, but it may also increase the risk of nocebo effect, where suboptimal clinical outcomes occur due to negative expectations and not the treatment itself.^15–17^

To the best of our knowledge, patient knowledge and perceptions toward biosimilars have not yet been investigated in Greece. Therefore, the objective of this study was to assess patients’ understanding and attitudes about biosimilars in rheumatoid diseases in Greece.

## METHODS

### Design

This was a cross-sectional, observational study, which was conducted in Greece between January 2022 and September 2022. A convenience sample of patients with rheumatoid diseases who were registered members of the Panhellenic Federation of Associations of Patients, Parents, Guardians and Friends of children with rheumatic diseases (RHEUMAZIN) participated in this survey. All members of the patient group meeting the eligibility criteria were invited to complete the questionnaire. RHEUMAZIN is the largest group of patients with rheumatic diseases in Greece and respondents were recruited from different areas of the country. Participants had to be at least 18 years old, mentally capable to respond to the questionnaire, and were required to provide informed consent. Furthermore, respondents did not have to be treated with a biological agent at the time of the recruitment to be included in the study.

### Data collection

Data collection was carried out via an anonymous web-based questionnaire. The survey instrument was designed based on a comprehensive review of the literature and in collaboration with rheumatologists and the patient group. The questionnaire was pilot-tested in three patient interviews before the launch of the main fieldwork and it was adjusted according to their comments and suggestions. Information was collected on sociodemographics (sex, age, education level, and employment status), clinical characteristics (e.g. duration of disease, duration of biological therapy, and type of biological therapy), patients’ sources of information, and knowledge and attitudes towards bio-originators and biosimilars.

### Statistical analysis

Descriptive statistics were used to summarise patient sociodemographic and clinical characteristics. The two-sample test of proportions was used to examine the equality of the proportions of people who “agreed” or “strongly agreed” with each perception statement between the respondents that had and those who had not knowledge about biosimilars. Also, the significance of the difference between these two groups with regards to the average level of patient concern (0–10 scale) if they switched to a biosimilar treatment was checked by the Mann–Whitney U test. The univariate associations between various sociodemographic, clinical and other patient characteristics and patient positive view towards biosimilars were explored with unadjusted odds ratios (OR), which were calculated using multiple binary logistic regressions.

The level of significance was set at α = 0.05. Statistical analysis was performed using STATA® v.17.

## RESULTS

### Survey participants

A total of 309 patients with rheumatoid diseases participated in this study. Patient sociodemographic and clinical characteristics are presented in **Table 1**. The vast majority of the respondents were females (73.8%), and the mean age was 54.1 (±12.5) years. Over half of the sample had a tertiary education (52.8%) and 49.2% of the respondents were employed at the time of the study. Most patients had been diagnosed with a rheumatic disease for more than 10 years (68.6%) and reported having good or very good general health (55.3%). Furthermore, 65.5% of the participants were taking a biologic agent for at least 6 years. Bio-originators was the main type of biological therapy (60.2%), while the share of biosimilars was only 11% and 28.8% of the patients did not know what type of biological therapy were taking. About 15.1% of the respondents had received treatment with a biosimilar at some point since their diagnosis, whereas 28.8% were not sure if they had ever been treated with a biosimilar.

**Table 1.** Sample characteristics.

**Variables**	**Categories**	**n**	**%**
Sex	Female	228	73.8
	Male	81	26.2
Age, mean (SD)		54.1 (12.5)	
	<50 years	117	37.9
	50–64 years	110	35.6
	65+ years	82	26.5
	Low education	146	47.2
Education level			
	High education	163	52.8
	Working (e.g. employee, self-employed)	152	49.2
Employment status	Not working (e.g. at home, unable to work, student, unemployed)	72	23.3
	Retired	85	27.5
	1–9 years	97	31.4
Years since diagnosis	10–19 years	133	43.0
	20+ years	79	25.6
	0–2 years	49	16.0
	3–5 years	57	18.6
Years taking biological therapy			
	6–10 years	97	31.6
	11+ years	104	33.9
	Bio-originator	186	60.2
Type of biological therapy	Biosimilar	34	11.0
	Don't Know	89	28.8
	No	172	56.4
Ever received treatment with biosimilar?	Yes		15.1
		46	
	Don't know/Not sure	87	28.5
	Very good/good	171	55.3
General health	Fair	96	31.1
	Bad/very bad	42	13.6

### Perceived knowledge and sources of information

**Figure 1.**
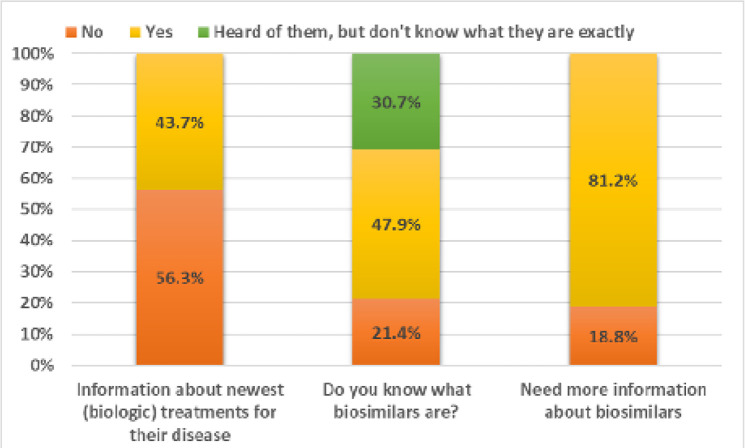
Self-perceived knowledge about biologic treatments.

**Figure 2.**
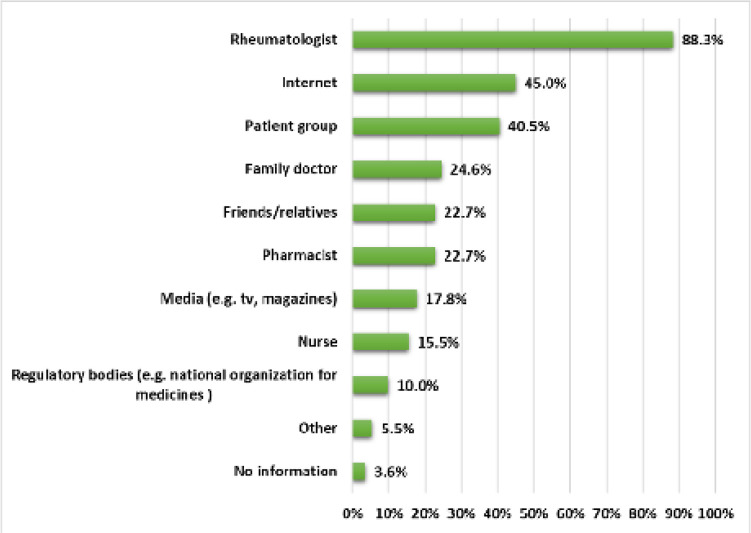
Main patients’ sources of information about biologic treatments.

Out of the 309 participants, 43.7% reported they had adequate information about the newest (biologic) treatments for their disease (**Figure 1**). However, only 47.9% knew what the term “biosimilar” referred to, whereas 30.7% were aware of their existence without knowing what they are exactly and 21.4% had never heard of biosimilars. Notably, 81.2% of the sample felt that they need more information about biosimilars. The main sources of information about biologic treatments were their rheumatologist (88.3%), the internet (45%) and patient associations (40.5%), followed by their family doctor (24.6%), friends and relatives (22.7%) and pharmacists (22.7%) (**Figure 2**).

### Patients’ attitudes and perceptions about biosimilars

**Figure 3.**
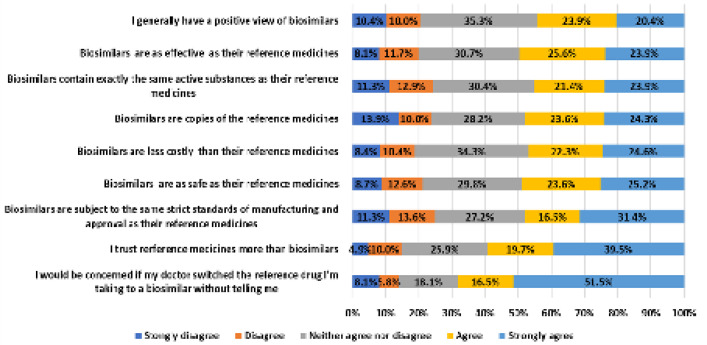
Patients’ perceptions of biosimilars.

When asked about their perceptions of biosimilars, 44% of the participants reported having a generally positive view of them, although 60% of the patients trusted more the reference biological products (**Figure 3**). Almost half of the sample thought that biosimilars are as effective (50%) and safe (49%) as the bio-originator products and fewer than half of the patients (47%) agreed that they are less costly. Moreover, 48% and 45% of the respondents believed that biosimilars are copies of and that they contain exactly the same active substances as their reference medicines, respectively. Less than half of the sample (48%) agreed that biosimilars are subject to the same strict standards of manufacturing and approval as their reference medicines. Also, about two thirds of the sample (68%) would be concerned if their physician switched the bio-originator product to its biosimilar without first discussing it with them.

**Figure 4.**
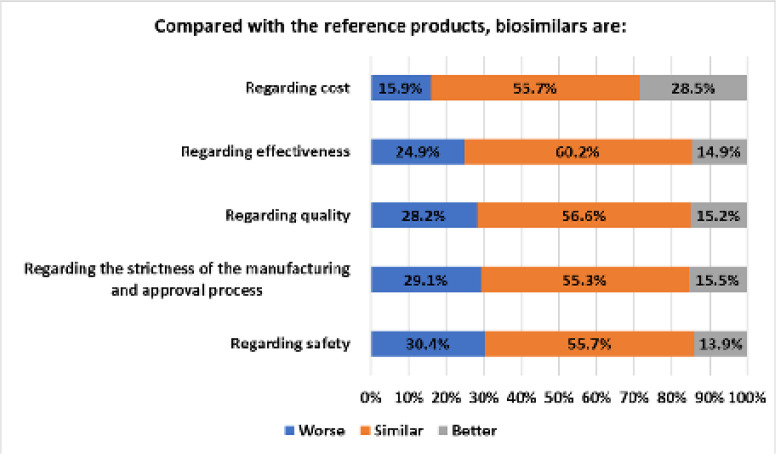
Patients’ subjective comparison of bio-originator products with biosimilars.

Subsequently, respondents were asked to compare biosimilars with bio-originator products (**Figure 4**). Patients were mostly concerned about the relative safety of biosimilars, with 30.4% considering them worse than their reference counterparts with respect to side-effects. Also, 29.1% and 28.2% of the sample thought that biosimilars are inferior to the bio-originator products in terms of the standards of the manufacturing and approval process and quality, respectively. About 24.9% of the participants were concerned that biosimilars are less effective than the reference medicines and, interestingly, 15.9% believed that biosimilars are more costly.

When the share of respondents who “agreed” or “strongly agreed” with each perception statement about biosimilars was compared between the respondents that had and those who had not knowledge about biosimilars, some perception gaps were identified (**Table 2**). All perception gaps numerically favoured the group of patients who reported they had knowledge about biosimilars. However, a statistically significant difference was not established for the items of trusting reference medicines more than biosimilars and being concerned if their doctor switched the reference product to a biosimilar without telling them. The widest gap was related to the manufacturing and approval conditions (−66.3%, p-value<0.001), followed by effectiveness (64.5%, p-value<0.001), and safety (64.4%, p-value<0.001).

**Table 2.** Knowledge about biosimilars and perception gap.

**Perceptions+**	**Have knowledge about biosimilars**		**Perception gap**	**p-value‡**

	No*	**Yes**		
Biosimilars are copies of the reference medicines	21.1%	77.0%	−55.9%	<0.001
Biosimilars contain exactly the same active substances as their reference medicines	16.8%	76.4%	−59.6%	<0.001
Biosimilars are subject to the same strict conditions of manufacturing and approval as their reference medicines	16.1%	82.4%	−66.3%	<0.001
Biosimilars are as effective as their reference medicines	18.6%	83.1%	−64.5%	<0.001
Biosimilars are as safe as their reference medicines	18.0%	82.4%	−64.4%	<0.001
Biosimilars are cheaper than their reference medicines	38.5%	56.1%	−17.6%	0.002
I would be concerned if my doctor switched the reference drug to a biosimilar without telling me	66.5%	69.6%	−3.1%	0.555
I trust reference medicines more than biosimilars	57.8%	60.8%	−3.0%	0.586
I generally have a positive view of biosimilars	18.0%	73.0%	−55.0%	<0.001
How concerned would you be if you switched to a biosimilar, mean(0–10 scale)	7.62	5.58	2.04	<0.001†

+ The responses “agree” and “strongly agree” were combined for each statement.

* Respondents were classified as “No” if they answered “No” or “I've heard of them but I don't know what they are exactly”.

‡ The two-sample test of proportions was used to examine the equality of the proportions.

† The significance of the difference between the two groups was checked by the Mann–Whitney U test.

### Factors associated with patient positive view of biosimilars

**Table 3** presents the results of the univariate analysis of factors associated with patient positive view of biosimilars. High education level (OR: 2.91, 95%CI: 1.82–4.65), previous treatment with biosimilar (OR: 3.86, 95%CI: 1.80–8.27), having rheumatologists (OR: 3.14, 95%CI: 1.38–7.13), patient associations (OR: 2.95, 95%CI: 1.84–4.73), regulatory bodies (OR: 5.01, 95%CI: 2.09–12.01) and the internet (OR: 2.16, 95%CI: 1.36–3.41) as main sources of information, being fairly (OR: 3.20, 95%CI: 1.04–9.90) or well-informed (OR: 8.49, 95%CI: 2.84–25.38) about their illness, being informed about the newest biological therapies (OR: 1.93, 95%CI: 4.50–12.39) and biosimilars (OR: 27.00, 95%CI: 10.82–67.36) or having heard of them (OR: 3.19, 95%CI: 1.22–8.36) were significantly associated with a higher likelihood of patients having a positive view of biosimilars. Not working (OR: 0.36, 95%CI: 0.20–0.65) or being retired (OR: 0.46, 95%CI: 0.27–0.80), not knowing if had been treated with biosimilars in the past (OR: 0.24, 95%CI: 0.13–0.45) and needing more information about them (OR: 0.49, 95%CI: 0.28–0.88) were decreasing the odds of having a positive view of biosimilars.

**Table 3.** Univariate associations between potential factors and patient positive view about biosimilars.

**Variables**	**Categories**	**Unadjusted Odds Ratio**	**95% CI**		**p-value**
Sex (ref.: male)	Male	1.61	0.97	2.69	0.066
Age (ref.: <50 years)	50–64 years	0.76	0.45	1.28	0.306
	65+ years	0.57	0.32	1.01	0.054
Education level (ref.: low level)	High level	2.91	1.82	4.65	<0.001
Employment status (ref.: working)	Not working (e.g. at home)	0.36	0.20	0.65	0.001
	Retired	0.46	0.27	0.80	0.006
Years since diagnosis (ref.: 1–9 years)	10–19 years	0.79	0.47	1.34	0.381
	20+ years	1.07	0.59	1.94	0.821
Years taking biologic therapy (ref.: 0–2 years)	3–5 years	0.99	0.46	2.13	0.988
	6–10 years	0.80	0.40	1.59	0.518
	11+ years	0.55	0.28	1.10	0.091
Previous treatment with biosimilar (ref.: no)	Yes	3.86	1.80	8.27	0.001
	Don't know/Not sure	0.24	0.13	0.45	<0.001
Source of information (ref.: no)	Family doctor	1.45	0.86	2.44	0.159
	Rheumatologist	3.14	1.38	7.13	0.006
	Pharmacist	1.25	0.73	2.13	0.418
	Patient group	2.95	1.84	4.73	<0.001
	Nurse	1.60	0.86	2.96	0.138
	Media	1.51	0.84	2.71	0.169
	Regulatory bodies	5.01	2.09	12.01	<0.001
	Internet	2.16	1.36	3.41	0.001
	Friends/relatives	1.34	0.79	2.29	0.279
Knowledge about illness (ref. poorly informed)	Fairly informed	3.20	1.04	9.90	0.043
	Well informed	8.49	2.84	25.38	<0.001
Knowledge about newest (biologic) therapies (ref.: no)	Yes	1.93	4.50	12.39	<0.001
Knowledge about biosimilars (ref.: no)	Yes	27.00	10.82	67.36	<0.001
	Heard of them, but don't know what they are exactly	3.19	1.22	8.36	0.018
Need more information about biosimilars (ref.: no)	Yes	0.49	0.28	0.88	0.016

## DISCUSSION

To the best of our knowledge, this is the first study assessing patients’ perceptions of biosimilars in rheumatology as well as biosimilars in general in Greece.

Patient advocacy groups provide support and education for their members. Therefore, it is reasonable to expect that patients participating in such patient associations can fill their information gaps about their disease and treatment options not met by their healthcare providers through education programmes provided by the patient groups.^18^ Nevertheless, although all respondents of this survey were members of a rheumatoid patients’ association, the majority reported having inadequate knowledge about the biologic therapies. Also, no more than half of them knew what biosimilar drugs are exactly and more than 80% of the sample felt that they need more information about biosimilars. In fact, almost three in every ten patients were not aware about the type of biologic therapy they were receiving at the time of the interview or if they had ever been treated with a biosimilar. Taken together, these findings indicate that unmet information needs about biologic therapies and their biosimilars still remain among rheumatoid patients in Greece. Furthermore, since all respondents were members of a patient association, the actual understanding level of biosimilars may be even lower in non-member patients as well as in the general population. In a study conducted in the US and the European Union, only 6% of the general population and about 20% and 30%, respectively, of members of patient advocacy groups (not restricted to rheumatology) reported at least a general awareness of biosimilars, reported at least a general of biosimilars.^9^ In another study among rheumatoid patients in France, 43% of the sample knew what biosimilars were.^2^

The low patient knowledge and understanding of biosimilars is creating information gaps that negatively affect their trust on biosimilars and their predisposition to be prescribed biosimilar treatment. According to our results, the main sources of patient information about biologic treatments were rheumatologists, the internet and patient associations. Previous research has shown that rheumatologists provide patients with selective information about biosimilars before suggesting switching from the reference biologic products, since they fear that patients’ concerns may increase.^19^ However, other surveys have demonstrated that such information could reassure patients and boost their willingness to switch to biosimilar treatment.^2^

Several misconceptions about biosimilars that should be educational/informational topics discussed by healthcare providers and patient advocacy groups with patients were identified by this survey. Only about 55–60% of the participants thought that biosimilars are comparable to their reference biological products in terms of safety, effectiveness, quality and the standards of the manufacturing and approval process. Notably, there were several patients (around 14–16%) that considered biosimilars better than bio-originators in the previously mentioned dimensions, while 16% of the sample also believed that biosimilars are more costly.

The information gaps among respondents lead to significant perception differences between those with and those without a basic understanding of biosimilar products. The widest perception gaps between these groups were established with respect to the regulatory requirements, effectiveness and safety of biosimilars relative to the reference products. Overall, the size of these gaps was found to be more than three times larger than in a previous study conducted in the European Union and the US.^9^

Previous research has shown that fear of loss of control of rheumatoid arthritis and adverse events are major patient concerns, which makes them reluctant to change their treatment.^20^ These risks are perceived even less acceptable in the case of switching from a bio-originator to its biosimilar.^2^ In addition to being emotionally attached to their treatment, patients do not have any personal or clinical interest in switching to biosimilar treatment.^2^ In a study in French rheumatoid patients, only 15% would accept to change their bio-originator treatment to its biosimilar without worrying.^2^ However, our survey showed that the magnitude of concerns is much higher in patients not having an adequate understanding of biosimilars.

Less than half of the participants reported having a generally positive view of biosimilars and the majority stated they placed more trust in the reference biological products. A higher education level, previous experience with biosimilar treatment, having rheumatologists, patient associations, regulatory bodies and the internet as main sources of information, being better informed about the disease, the newest biological therapies and biosimilars, working and having adequate information about biosimilars were univariately associated with a significantly higher likelihood of patients having a positive view of biosimilars.

Overall, the abovementioned findings indicate that patients are in need of some basic background information about the regulatory requirements for demonstration of comparable efficacy and safety with the reference biological product that should be met for a biosimilar to be approved.^9^ Also, patients should be better informed about how biosimilars can offer significant costs savings to patients and public and private insurance plans, thus improving patient access and the sustainability of healthcare systems, but still delivering the same value as the bio-originator products.^9,21^ There is a common preconception among patients that lower cost indicates lower quality.^20,22^ Interestingly, in our study, the degree of trust in biosimilars relative to their reference products was not found to be significantly different with respect to the subjective level of patient knowledge. Therefore, any reservations that patients may have about taking biologic treatment in general should also be addressed, since this scepticism may render them reluctant to take biosimilars for the reason that they are biologic medicines.^9^ Finally, education programs regarding biologic therapies and biosimilars should also target caregivers, since their perceptions closely reflect and are intertwined with those of the patients to which they provide help.^9^

This study had some limitations that should be taken into account while interpreting its results. A convenience sample of patients who were members of the RHEUMAZIN patient group was utilised for the purpose of this study, which may not have been as representative as a sample selected using a probabilistic sampling method would have been. However, this rheumatoid patient association is the largest in the country and it was formed as a federation of other smaller patient groups located in different regions of the country. Moreover, the questionnaires were web-based and no face-to-face interviews were conducted, which may have introduced some measurement error. Nevertheless, clarifications were provided by the investigators if requested. Finally, whether patients had an adequate understanding of biosimilars was not measured objectively, rather it was based on respondents’ self-assessment.

## CONCLUSION

There appears to exist a great need for education about biosimilars across rheumatoid patients, since several gaps in knowledge and false perceptions about the use of biosimilars in rheumatology were identified in this study. Health education programs are required to provide patients with the necessary information to make informed decisions about their treatment and the use of biosimilars. These programs should be developed by healthcare providers in partnership with patient associations. Patient associations are considered important contributors to patient education and may help educate patients who are already more engaged. Nevertheless, such partnerships would benefit all patients, regardless of their level of involvement with patient associations, through patient education materials that better reflect the information needs of patients.

## References

[1] Quinlivan A Lester S Barrett C Whittle S Rowett D Black R Attitudes of Australians with inflammatory arthritis to biologic therapy and biosimilars. Rheumatol Adv Prac 2022;6. 10.1093/rap/rkac099 PMC9682816 36424984

[2] Frantzen L Cohen JD Tropé S Beck M Munos A Sittler MA Patients’ information and perspectives on biosimilars in rheumatology: A French nation-wide survey. Joint Bone Spine 2019;86:491–6. 30659920 10.1016/j.jbspin.2019.01.001

[3] Bonafede M Joseph GJ Princic N Harrison DJ. Annual acquisition and administration cost of biologic response modifiers per patient with rheumatoid arthritis, psoriasis, psoriatic arthritis, or ankylosing spondylitis. J Med Econ 2013;16:1120–8. 23808901 10.3111/13696998.2013.820192

[4] Al Maini M Adelowo F Al Saleh J Al Weshahi Y Burmester GR Cutolo M The global challenges and opportunities in the practice of rheumatology: white paper by the World Forum on Rheumatic and Musculoskeletal Diseases. Clin Rheumatol 2015;34:819–29. 25501633 10.1007/s10067-014-2841-6 PMC4408363

[5] van Overbeeke E De Beleyr B de Hoon J Westhovens R Huys I. Perception of Originator Biologics and Biosimilars: A Survey Among Belgian Rheumatoid Arthritis Patients and Rheumatologists. BioDrugs 2017;31:447–59. 28929342 10.1007/s40259-017-0244-3

[6] Varma M Almarsdóttir AB Druedahl LC. “Biosimilar, so it looks alike, but what does it mean?” A qualitative study of Danish patients' perceptions of biosimilars. Basic Clin Pharmacol Toxicol 2022;130:581–91. 35261174 10.1111/bcpt.13719 PMC9314148

[7] Giuliani R Tabernero J Cardoso F McGregor KH Vyas M de Vries EGE. Knowledge and use of biosimilars in oncology: a survey by the European Society for Medical Oncology. ESMO Open 2019;4:e000460. 30962961 10.1136/esmoopen-2018-000460 PMC6435239

[8] Teeple A Ginsburg S Howard L Huff L Reynolds C Walls D Patient attitudes about non-medical switching to biosimilars: results from an online patient survey in the United States. Cur Med Res Opin 2019;35:603–9. 10.1080/03007995.2018.1560221 30618353

[9] Jacobs I Singh E Sewell KL Al-Sabbagh A Shane LG. Patient attitudes and understanding about biosimilars: An international cross-sectional survey. Patient Prefer Adherence 2016;10:937–48. 27307714 10.2147/PPA.S104891 PMC4889091

[10] Cohen H Beydoun D Chien D Lessor T McCabe D Muenzberg M Awareness, Knowledge, and Perceptions of Biosimilars Among Specialty Physicians. Adv Ther 2017;33:2160–72. 27798772 10.1007/s12325-016-0431-5 PMC5126187

[11] Chen AJ Gascue L Ribero R Van Nuys K. Uptake of Infliximab Biosimilars Among the Medicare Population. JAMA Internal Med 2020;180:1255–6. 32702080 10.1001/jamainternmed.2020.3188 PMC7372498

[12] Kim Y Kwon HY Godman B Moorkens E Simoens S Bae S. Uptake of Biosimilar Infliximab in the UK, France, Japan, and Korea: Budget Savings or Market Expansion Across Countries? Front Pharmacol 2020;11:970. 32733238 10.3389/fphar.2020.00970 PMC7363801

[13] Yfantopoulos JN Chantzaras A. Drug Policy in Greece. Value Health Reg Issues 2018;16:66–73. 30195093 10.1016/j.vhri.2018.06.006

[14] Papachristos A Kani C Litsa P Valsami G Souliotis K Saridi M Drug utilization patterns and costs of erythropoiesis-stimulating agents in an outpatient setting in Greece. Consult Pharm 2016;31:271–81. 27178657 10.4140/TCP.n.2016.271

[15] Gasteiger C Scholz U Petrie KJ Dalbeth N. A bio-what? Medical companions’ perceptions towards biosimilars and information needs in rheumatology. Rheumatol Int 2022;42:1993–2002. 34705051 10.1007/s00296-021-05037-5

[16] Rezk MF Pieper B. Treatment Outcomes with Biosimilars: Be Aware of the Nocebo Effect. Rheumatol Ther 2017;4:209–18. 29032452 10.1007/s40744-017-0085-z PMC5696297

[17] Pouillon L Socha M Demore B Thilly N Abitbol V Danese S The nocebo effect: a clinical challenge in the era of biosimilars. Exp Rev Clin Immunol 2018;14:739–49. 10.1080/1744666X.2018.1512406 30118338

[18] Nijsten T Bergstresser PR. Patient advocacy groups: let's stick together. J Invest Dermatol 2010;130:1757–9. 20548309 10.1038/jid.2010.131

[19] Waller J Sullivan E Piercy J Black CM Kachroo S. Assessing physician and patient acceptance of infliximab biosimilars in rheumatoid arthritis, ankylosing spondyloarthritis and psoriatic arthritis across Germany. Patient Prefer Adherence 2017;11:519–30. 28331299 10.2147/PPA.S129333 PMC5356924

[20] Wolfe F Michaud K. Resistance of rheumatoid arthritis patients to changing therapy: discordance between disease activity and patients' treatment choices. Arthritis Rheum 2007;56:2135–42. 17599730 10.1002/art.22719

[21] Kvien TK Patel K Strand V. The cost savings of biosimilars can help increase patient access and lift the financial burden of health care systems. Semin Arthritis Rheum 2022;52:151939. 35027243 10.1016/j.semarthrit.2021.11.009

[22] Aladul MI Fitzpatrick RW Chapman SR. Patients’ Understanding and Attitudes Towards Infliximab and Etanercept Biosimilars: Result of a UK Web-Based Survey. BioDrugs 2017;31:439–46. 28752242 10.1007/s40259-017-0238-1

